# Mental Health and Service Use of Migrants in Contact with the Public Psychiatry System in Paris

**DOI:** 10.3390/ijerph17249397

**Published:** 2020-12-15

**Authors:** Andrea Tortelli, Florence Perquier, Maria Melchior, François Lair, Fabien Encatassamy, Chloé Masson, Hélène K’ourio, Raphaël Gourevitch, Alain Mercuel

**Affiliations:** 1 Pôle Psychiatrie Précarité, Groupe Hospitalier Universitaire Paris Psychiatrie & Neurosciences, 75014 Paris, France; f.cslair@gmail.com (F.L.); CH.MASSON@ghu-paris.fr (C.M.); A.MERCUEL@ghu-paris.fr (A.M.); 2 Pierre Louis Institute for Epidemiology and Public Health (IPLESP/INSERM UMR_S 1136), 75012 Paris, France; maria.melchior@inserm.fr; 3 Institut des Migrations, 75013 Paris, France; 4 Departement d’Epidémiologie, Groupe Hospitalier Universitaire Paris Psychiatrie & Neurosciences, 75014 Paris, France; fperquier@gmail.com; 5 CPOA: Centre Psychiatrique d’Orientation et d’Accueil, Hôpital Sainte-Anne, Groupe Hospitalier Universitaire Paris Psychiatrie & Neurosciences-1, rue Cabanis, F-75014 Paris, France; fabien.encatassamy@gmail.com (F.E.); helene.kourio@ch-montperrin.fr (H.K.); R.GOUREVITCH@ghu-paris.fr (R.G.)

**Keywords:** migrants, mental health, mental health care provision, asylum seekers, vulnerable groups

## Abstract

Background: Migrants, and particularly asylum seekers, are at increased risk of psychiatric disorders in comparison with natives. At the same time, inequalities in access to mental health care are observed. Methods: In order to evaluate whether the Parisian public psychiatric system is optimally structured to meet the needs of this population, we examined data on mental health and service use considering three different levels: the global system treatment level, a psychiatric reception center, and mobile teams specializing in access to psychiatric care for asylum seekers. Results: We found higher treatment rates among migrants than among natives (*p* < 0.001) but inequalities in pathways to care: more mandatory admissions (OR = 1.36, 95% CI: 1.02–1.80) and fewer specialized consultations (OR = 0.56, 95% CI: 0.38–0.81). We observed a mismatch between increased need and provision of care among migrants without stable housing or seeking asylum. Conclusions: Inequalities in the provision of care for migrants are observed in the Parisian public psychiatric system, particularly for those experiencing poor social and economic conditions. There is a need to facilitate access to mental health care and develop more tailored interventions to reduce discontinuity of care.

## 1. Introduction

Migrants and ethnic minorities are at increased risk of psychiatric disorders in comparison with natives, mostly due to cumulative exposure to psychosocial risk factors before (war, economic and political crisis, persecution), during (exposure to violence, food insecurity, risk of death), and after migration (discrimination, resettlement stress, social adversity) [1]. For instance, asylum seekers and refugees are at higher risk of serious mental health outcomes than economic migrants [2,3], especially those who have seen their asylum application rejected [4].

At the same time, inequalities in access to mental health care are observed, as a result of many barriers which include unstable housing, lack of access to welfare, language barriers, lack of knowledge of the health care system, differences in perception of health and in help-seeking behaviors, and experience of stigma [5,6,7,8].

Recently, migration following conflicts in the Middle East and Africa has resulted in an increase in mental health care needs in host countries and has contributed to revealing gaps in the mental health provision for this population particularly exposed to stressful factors [9]. Public and private mental health care systems have been quickly overloaded, leading migrants to often have their first contact with the mental health care system through emergency departments [10,11,12].

In France, as a result of many waves of migration over several decades, migrants account for 11% of the general population, and most live in the Paris region [13]. Recently, as observed in many European countries, a growing number of migrants have seen their living conditions worsen due to restrictive immigration policies, leading to social vulnerability [14,15,16]. In 2019, only 26% of 138,000 asylum seekers’ applications were granted refugee status. The Paris region is home to around 60% of all undocumented migrants living in France (estimated to be approximately 300,000 before 2012) and one in two homeless persons in Paris was born abroad [17].

Epidemiological studies in general population samples in France showed an increased prevalence of mood disorders and psychosis in migrants compared with natives [18,19]. Regarding migrants with poor living conditions, two studies conducted in the Greater Paris area showed increased vulnerability to psychiatric disorders compared to the general population. The “Enfants et FAMilles sans logement personnel en Ile-de-France Study”—ENFAMS (Characteristics and health of homeless families)—was conducted among families sheltered in social accommodation, with more than nine in ten parents being born outside of France [20]. The prevalence of depression was 30% among mothers, and psychological distress was found in approximately 20% of children [21]. Factors associated with women’s poor mental health included exposure to violence during migration, as well as food and administrative insecurity in France.

A second study, “La Santé Mentale et les addictions chez les personnes sans logement personnel d’Ile-de-France”—SAMENTA (Prevalence of Mental Disorders and Addictions among Homeless People)—was conducted among homeless people and found three times more depressive episodes and six times more post-traumatic stress disorder (PTSD) among migrants in comparison with natives [22].

Regarding access to mental health care, barriers have been observed in relation to poor living conditions [23]. In the ENFAMS study, 25% of mothers did not seek care for economic reasons (lack of income or insurance). In the SAMENTA study, among people with severe mental health problems (psychosis, severe mood disorders, and anxiety), only 30% of migrants had a psychiatric follow-up compared to 55.6% of natives. Furthermore, a recent survey conducted also revealed that challenges in the collaboration and cooperation between mental health and social service providers may result in unmet mental health care needs of migrants living in social accommodation [24].

### The Public Mental Health Care System in France

The French mental health care system is organized by geographical subunits (“secteurs psychiatriques”), to which people are assigned based on their place of residence. It ensures easy access and continuity of care for all inhabitants, wherever they live [25]. Each of these subunits includes inpatient and outpatient facilities and has a catchment area of around 60,000 to 80,000 inhabitants. For people without stable housing, the structure of reference usually corresponds to that of the geographical area where they spend most of their time. For persons with chronic or severe mental disorders, access to care is free, except for ward costs (17 euros/day), which need to be covered by private assurance. Regarding migrants, disparities in mental health care provision by the public system exist due to two main reasons. First, there is a widespread belief among mental health specialists in France regarding the need of specific knowledge about migrant psychology/psychopathology to treat this population. Initially theorized by colonial psychiatry, and then by specialists of psychoanalytic trauma theories, this assumption excludes migrants from usual care delivered by professionals from the public system, which has been shaped in line with republican values of equality and universality [26]. Therefore, migrants tend to be referred to private “specialized” psychosocial centers or non-governmental organizations (NGOs). However, these centers provide, essentially, counselling and psychotherapy, with limited capacity, and they lack coordination with the public mental health care system, thus not meeting the needs of migrants with more severe psychiatric disorders, who may need hospitalization or pharmacological treatment. The second obstacle is political. As discussed above, restrictive migration policies have worsened the living conditions of this population, adding two important barriers to access and continuity of care: unstable housing and restricted or non-existent access to welfare benefits [27]. For instance, undocumented migrants have access to inpatient and outpatient care owing to the “Aide Médicale Etat-AME”—state medical insurance. However, this population does not have access to specialized housing facilities or to the AAH—Allocation Adulte Handicapé (disability allowance)—hindering the possibility for those with severe disabilities to benefit from social services support and adapted mental health care after hospital discharge.

Considering that, in 2016, mental health problems accounted for nearly 11% of the burden of non-communicable diseases in France [28] and that migrants are likely to be at increased risk compared to natives, we sought to describe the mental health and service use of the migrant population attending various psychiatric mental health care facilities in the Paris area, with a focus on those without stable housing or seeking asylum. To meet this aim, we gathered descriptive data from mental health services attended by migrants, taking into account three different levels: the global treatment level, a psychiatric reception center, and mobile teams specializing in access to psychiatric care for asylum seekers. As far as we know, there is no other study on this subject, and we hope that ours will contribute to the design of more tailored mental health care interventions for this population.

## 2. Materials and Methods

This article describes results from four cross-sectional studies carried out in different facilities of the Parisian public psychiatric system.

### 2.1. The “Santé Mentale et Logement” (Mental Health and Housing) Survey

This study was carried out on 31 January 2017 in three psychiatric hospitals serving the population living in Paris: the University Hospital Group for Psychiatry & Neurosciences (GHU-Paris), the National Hospital of Saint-Maurice, and the Mental Health Association of the 13th arrondissement in Paris (ASM13). All patients aged over 18 present or seen in inpatient and outpatient care units, except those treated in emergency services, were eligible to participate. Information regarding demographics, socio-economic situation, medical insurance coverage, and contact with a general practitioner were collected. Psychiatric diagnoses were coded using the 10th International Classification of Mental and Behavioural Disorders (ICD-10). Data on mental health care services use (inpatient and outpatient care facilities, ad hoc specialized consultation, mandatory admissions) were obtained based on where the patient was present or seen on the corresponding day. Patients were categorized according to their place of birth as native (born in France) or migrant (born abroad). Day-point estimates of treatment rates for mental disorders were calculated in reference to data from the French census on the adult population living in Paris in 2017. We examined differences between native and migrant patients with regard to service utilization and socioeconomic, housing, and medical characteristics, using chi-square tests and odds ratios and their 95% confidence intervals (STATA 14, StataCorp LLC, College Station, TX, USA).

### 2.2. Migrants without Stable Housing Seen at the Reception and Guidance Psychiatric (Centre Psychiatrique d’Orientation et d’Accueil—CPOA)

The CPOA is a psychiatric consultation (around 10,000/year) offering evaluation and referral to psychiatric facilities for all people living in the Paris area, especially designed to attribute a sector to people without stable housing, estimated to be around 20% in recent years. A two-month survey carried out in 2017 using data from caseloads of migrants without stable housing analyzed the prevalence of psychiatric disorders (ICD-10), as well referral and follow-up rates.

### 2.3. Asylum Seekers

The last two surveys rely on the analysis of data collected from psychiatric consultations for asylum seekers conducted by mobile psychiatric teams: Equipe Mobile Psychiatrie Précarité—EMPP (Mobile psychiatric-precariousness teams) [29].

The first one was based on a one-year study in 2018 in social accommodation for recently arrived migrant families (Centre d’Hebergement d’Urgence pour Migrants—CHUM) in Ivry, a southern suburb of Paris. Prevalence rates of psychiatric disorders were ascertained using the DSM-V Diagnostic and Statistical Manual of Mental Disorders, fifth edition).

The second one was based on a 9-month study carried out in 2019 in a reception center (Centre d’Accueil et d’Evaluation des Situations—CAES) for adult men (>18 years), in the 18th district (in Northern Paris). In this setting, only one consultation per patient was possible; therefore, we sought to identify the presence of severe psychiatric symptoms (delusions, hallucinations, suicidal ideas) rather than establish a diagnosis, except for PTSD, which was ascertained using the PTSD-8 scale [30].

In both settings, face-to-face interpreter services were extensively used.

## 3. Results

### 3.1. Mental Health Care Use among Migrants in the Parisian Public Psychiatric Facilities

In the “Santé Mentale et Logement” survey, 26.3% of 4005 patients present or seen in mental health care units on the day of the survey were migrants. Missing data were observed for 27 patients. Treatment rates were 2.15/1000 inhabitants in natives and 2.4/1000 inhabitants in migrants (*p* < 0.001). Migrants were more often men, single, and older than natives. They clearly experienced more socioeconomic disadvantages (Table 1).

Hospitalizations and mandatory admissions by police services were significantly more frequent among migrants compared to natives. Migrants were also more often diagnosed with psychosis and less often attended specialized consultations. They also had less access to medical insurance and less contact with general practitioners (Table 2).

### 3.2. Migrants without Stable Housing

In the CPOA, the two-month survey showed that migrants represented 25.0% of people without stable housing. They arrived for initial contact in 67.1% of cases, vs. 14.6% of natives. The main diagnoses were mood (41.1%) and anxiety (37.0%) disorders. Referral to outpatient facilities was made for 68.5% (vs. 34.1% natives). However, roughly only one in two patients attended mental health care elsewhere.

### 3.3. Asylum Seekers

In both surveys, patients mainly came from Afghanistan, Iraq, Somalia, or the Sudan.

In the social accommodation for families recently arrived in France, 93 patients (26 < 18 years, 61 women) attended the psychiatric consultation. Anxiety disorders were diagnosed in 59.1% of cases; mood disorders in 32.3%, and psychosis in 1.1%. Sixty per cent of children under 10 met criteria for a diagnosis of anxiety disorder and 6.7% of a mood disorder.

In the reception center for men, nearly 20% of residents requested to meet a psychiatrist, but only 1 out of 2 did actually attend the consultation (N = 131). Ninety-three patients (71.5%) showed severe psychiatric symptoms requiring referral to emergency ward (20%) or outpatient facilities: suicidal ideas were observed in 17.7% of cases, psychotic symptoms (delusions or hallucinations) in 10.0%, and 53.9% met criteria for a diagnosis of PTSD. Of note, 76.9% of patients with psychotic symptoms also had a diagnosis of PTSD. In this center, except for persons who were referred to emergency wards, there was no feedback regarding follow-up. Additionally, migrants who were denied refugee status or who fell under the Dublin regulation had no access to a shelter after their stay in the reception center, leading in most cases to homelessness and therefore increased risk of discontinuity of care.

## 4. Discussion

In this paper, we aimed to describe the mental health and service use of migrants in contact with the public psychiatric system in Paris. Overall, migrants had higher treatment rates in comparison to natives, especially for those with a diagnosis of psychosis and under mandatory admission. On the other hand, a lower level of utilization of specialized consultations was observed. Among migrants without stable housing or seeking asylum, an important mismatch between mental health care needs and the risk of unmet care was observed.

Our findings are in line with data from other European countries showing, in recent years, increased mental health care needs and inequalities in the provision of care for migrants in their host countries [31,32,33]. Social adversity has been put forward as one the main explanations [15,34], because it is associated not only with the development of psychiatric disorders [35,36] but also with barriers to access and continuity of care, in addition to well-known barriers such as language and cultural aspects [8]. In line with this observation, a recent study on ethnic variations in duration of untreated psychosis showed an association with disadvantaged social conditions and isolation but not with ethnicity [37]. In France, studies also showed that social adversity is related to worse health status [27] and is a barrier to mental health care [38,39].

As also observed across our data, discontinuity and underutilization of mental health care services seems to be a major concern and challenge for public services in countries hosting migrants. The same trend was found in studies conducted in similar settings. A study carried out in a reception center in Germany among 1533 residents, coming mostly from the Eastern Mediterranean region, showed rates of mental health care utilization of 47.5% among people with psychiatric disorders [40]. Another study in a reception center analyzed mental health care needs and service utilization: after three months, among 66.0% of the patients referred to psychotherapy, none attended any appointment, although their symptoms of psychiatric disorders remained in a clinically relevant range [41].

Regarding diagnosis, the increased treatment rates of psychosis are in line with previous data showing an increased risk of admission for psychosis among migrants [42], which is consistent with studies on migrants in the general population [18,43]. Moreover, in our study, this may also indicate lower access to mental health care among people presenting less severe psychiatric disorders, along with the barriers to care described above. Among asylum seekers, the prevalence of common mental disorders, PTSD, and psychotic symptoms is in line with studies showing an increased prevalence of mental health problems in comparison to the general population [2].

### 4.1. Limitations

Our data are essentially based on clinical records in mostly non-French-speaking samples. Although we used interpreters and standard diagnostic criteria to reduce diagnostic bias, due to the complexity of symptoms and contexts, misdiagnosis is still possible. Moreover, data on the chronicity of symptoms were not collected. Diagnosis may also have been impacted by the quality of clinical records and the limited number of consultations (particularly in the CAES). Finally, diagnostic overlap is possible, particularly between psychosis and PTSD, due to common clinical symptoms (dissociation, auditory hallucinations, blurred affect), as suggested in a growing number of studies [44,45].

Considering recruitment, bias cannot be ruled out, due to barriers with regard to access to a psychiatric consultation (cultural aspects, other appointments, short duration of stay in the reception center, help-seeking behaviors). Moreover, in the case of physical manifestations of depression, anxiety, or PTSD, referral to general practitioners instead of psychiatrists is possible, because these symptoms can co-occur with somatic complaints, especially among persons who have experienced physical violence [46].

Finally, based on the current literature, we may suppose that the increased prevalence of mental health problems in our samples is probably associated with adverse experiences related to the migration process, although in this study, we did not focus on these factors.

### 4.2. Clinical Considerations and Perspectives

Our findings embolden reconsideration of the current provision of mental health care for migrants by the public psychiatric system in France. There is a need to facilitate access and develop more tailored interventions, particularly for those living in deprived conditions or seeking asylum.

To this aim, one has to take into account the impact of social determinants of migrants’ health in regard not only to access but also to the prevention, development, and recovery of psychiatric disorders [47]. For instance, better conditions of resettlement are shown to be associated with better outcomes for mental health problems such as anxiety, depression, and even PTSD [48,49,50]. Therefore, notwithstanding immigration policies, which must facilitate access to the welfare state (including the health system), efficient mental health care for this population should be conceived through an integrative approach, with attention also given to living conditions [51,52].

In this sense, the quite explanatory model of migrants’ mental health problems developed by the ethnopsychiatry approach [53], which underscores the impact of culture with regard to the causes and treatment of ill mental health, in spite of the importance of the psychosocial environment [26,54], needs to be reconsidered. This may contribute to a more comprehensive understanding of the different risk factors related to the migratory experience and therefore a rethinking of the organization and access to effective care [55,56].

Barriers to care in migrants have been mainly related to help-seeking behaviors and lack of knowledge of the health care system [8,52,57]. Among mental health care providers, poor use of interpreters or lack of cultural competence are the main difficulties [58]. Moreover, a recent systematic review showed that the provision of healthcare services for migrants is deeply influenced by limited rights and low unfavorable social conditions, which may be incompatible with the use of prescribed medications, and with limited institutional capacity in terms of time and/or resources, which are in contradiction between physicians’ professional ethics and laws restraining access to care [6]. Barriers related to the extreme isolation of some groups such as unaccompanied minors could also worsen access to mental health care [59]. All these factors contribute to delay or unmet care characterized by increased use of emergency departments and ambulatory discharges, leading to an increased risk of deterioration of mental health conditions in the longer term [12,33].

To ensure effective integration and continuity of care in the psychiatric public system, mental health care organization should also be coordinated with referrals to other key professionals (general practitioners, social workers) [60]), particularly after the initial contact with the mental health system, and this should be extended to family members, including children [61]. Important attention needs to be given to the collaboration between mental health care specialists and primary care providers: many studies observed that migrants with mental health problems are more likely to seek primary care or attend emergency wards [46,62]. Similarly, strengthening collaboration between public and private providers could facilitate rapid access to complementary or intensive care [63,64].

Inequalities in the provision of mental health care for migrants in France are also observed regarding access to psychotherapy, migrants often being limited to psychoanalytical approach therapies, which may not be accessible or adequate for some groups. Brief psychotherapy, cognitive behavioral therapy (CBT), as well multidisciplinary treatments have proven their efficiency and effectiveness in the management of PTSD and common mental disorders and should be offered more broadly [65].

Moreover, there is a need to promote accessible interventions aiming to manage stress [66] and to support the attainment of social resources, such as language skills and social networks, which also contribute to reducing psychosocial distress and enhancing resilience [56,67]. For instance, counselling by peers supervised by mental health professionals has been successfully applied in places where access to mental health is limited and is being investigated in Europe, because it is cost-effective and culturally adapted [68,69].

Finally, research must continue to better identify migrants’ mental health care needs across patients of public and private providers, in different living and administrative conditions [51]. Moreover, research should be more frequently extended to diagnoses other than PTSD and common mental disorders. For instance, psychosis, the increased risk of which is well documented among migrants, may lead to significant vulnerability and burden. At the biological level, stress has been related to the development of mental health disorders [70,71,72], but studies among migrants are still lacking.

## 5. Conclusions

Within the public psychiatric system in Paris, one can observe differences in mental health care that affect migrants, especially in those without stable housing status, suffering from common mental disorders, or seeking asylum. Integrative approaches and increased collaboration between health and social services, and between public and private care providers, will contribute to boosting adequate access to mental health care and reducing exclusion from the public psychiatric system.

## Figures and Tables

**Table 1:** Sociodemographic characteristics of the sample, by migrant status.

Sociodemographic Characteristics	Migrant Status				Odds Ratio (95% CI)	
	Migrants N = 1055		Natives N = 2923			
	N	%	N	%		
**Sex**						
**Male**	609	57.7	1538	52.6	1.23	(1.06–1.42)
**Age**						
<30	127	12.0	495	16.9	0.66	(0.53–0.82)
≥60	239	22.7	572	19.6	1.20	(1.01–1.43)
Mean (sd)	47.8 (14.6)		45.9 (14.9)			
**Living conditions**						
Single	682	64.6	2276	77.9	0.51	(0.44–0.60)
Homelessness	74	7	44	1.5	4.93	(3.32–7.39)
Social accommodation	99	9.4	103	3.5	2.83	(2.10–3.81)
No income	130	12.3	108	3.7	3.66	(2.78–4.82)

**Table 2:** Diagnosis and service use, by migrant status.

	Migrant Status					
	Migrants N = 1055		Natives N = 2923			
	N	%	N	%	Odds Ratio	
					(95% CI)	
**Psychiatric disorders**						
Psychosis	617	58.5	1537	52.6	1.27	(1.09–1.46)
Mood disorders	173	16.4	536	18.3	0.87	(0.72–1.05)
Drug abuse	29	2.7	103	3.5	0.77	(0.49–1.19)
**Type of service**						
Inpatient facilities	343	32.5	836	28.6	1.20	(1.03–1.40)
Specialized consultation	37	3.5	177	6.1	0.56	(0.38–0.81)
Outpatient facilities	550	52.1	1482	50.7	1.05	(0.91–1.22)
**Mandatory admissions**	84	7.9	174	5.9	1.36	(1.02–1.80)
**Medical care**						
No general practitioner	320	30.3	520	17.8	2.01	(1.70–2.37)
No medical insurance	43	4.1	14	0.5	8.82	(4.71–17.53)
